# Dietary supplementing South African indigenous rams with flaxseed oil and ascorbic acid improves cryopreserved semen quality and in vitro fertility

**DOI:** 10.1007/s11250-024-04057-0

**Published:** 2024-07-10

**Authors:** Jabulani Nkululeko Ngcobo, Tshimangadzo Lucky Nedambale, Khathutshelo Agree Nephawe, Sindisiwe Mbali Sithole, Tlou Caswell Chokoe, Fhulufhelo Vincent Ramukhithi

**Affiliations:** 1 Department of Animal Sciences, Tshwane University of Technology, Private Bag X680, Pretoria, 0001 South Africa; 2 Agricultural Research Council, Germplasm, Conservation, Reproductive Biotechnologies, Private Bag X02, Irene, 0062 South Africa; 3 Department of Agriculture, Land Reform and Rural Development, Directorate Farm Animal Genetic Resource, Private Bag X250, Pretoria, 0001 South Africa

**Keywords:** Conservation, Docosahexaenoic acid, Long-chain Polyunsaturated fatty acids, Zulu, BaPedi, Namaqua Afrikaner

## Abstract

The purpose of this study was to evaluate how ascorbic acid with dietary flaxseed oil affects the quality and fertility of cryopreserved ram sperm in South African indigenous rams. Treatment diets were supplemented 60 days before semen collection to afford proper spermatogenesis, adaptation to the feed formulated and fed throughout the study. Semen was collected with the use of artificial vagina following dietary supplementation with five treatment diets (neg. cont. – negative control, pos. cont. – positive control, FLO – 5% Flaxseed oil, ASA – 4% Ascorbic acid, and FLO + ASA). Semen was then extended using tris-based extender and cryopreserved using the programmable freezer (CBS Freezer 2100 series, Laboratory consumables & chemical suppliers, America). Ovaries were collected from a neighbouring slaughter house and conveyed to the lab in 0.9% saline at 37 °C. Data (sperm parameters and in vitro fertility) was then exposed to the GLM (General Linear Model) in Minitab 17. Pearson’s correlation coefficient was utilized to investigate the relationship between cryopreserved sperm quality and in vitro fertility. The student Least Significant Difference Test was used to separate the treatment means, and differences were accepted when the *p*-value was less than 0.05. The FLO + ASA group had higher (*p* < 0.05) progressive (36.33 ± 1.87), total (88.24 ± 2.24), rapid motility (27.52 ± 1.74), intact plasma membrane (75.67 ± 2.08), total fertilization (65.98 ± 7.39), and total cleavage (66.19 ± 6.50) when compared to other treatment groups. Total fertilization rate had a medium significant (*p* < 0.001) medium correlation with the progressive motility (r2 = 0.435), total motility (r^2^ = 0.447) and rapid motility (r^2^ = 0.409). In conclusion, dietary flaxseed and ascorbic acid (FLO + ASA) improves cryopreserved semen quality, in vitro fertilization rate, and the total cleavage rate. Noteworthy, the progressive, total and rapid motility play a crucial in the in vitro fertilization rate.

## Introduction

Food insecurity remain a challenge in South Africa and globally (FAO 2022). According to Dlamini et al. (2023), about 20.4% of South African household are food insecure with the low socio-economic house being most affected. Domestic animals provide alternative source of protein to feed human beings (Kunene et al. 2009). The indigenous sheep of South Africa (Zulu, Bapedi, and Namaqua Afrikaner) are now threatened with extinction (Mazzera 2024). Their animal genetic materials can be conserved through cryopreserving semen for future use purposes and in vitro embryo production and ensuing embryo transfer (Mara et al. 2013; FAO 2014; Ngcobo et al. 2022).

Sperm cryopreservation is the most vital advanced reproductive biotechnologies in ruminants’ artificial insemination, because it allows the transport of semen from research stations to farms and allow the storage of semen for longer period of time (Leroy et al. 2019). Cryopreservation procedures and freezing mediums has been developed however, the frozen thawed small ruminants’ spermatozoa are still much lower than that of liquid stored (Salamon and Maxwell 2000). It is well known that only ± 60% of sperm cells remain viable and motile after cryopreservation (Lv et al. 2019) and only ± 30% remain biological functional (Salamon and Maxwell 2000). Furthermore, previous studies reported that cryopreserved sperm survival is not only dependent on species but also related to the individual and some environmental factors such as management and nutritional effects (Khan et al. 2021). For that reason, numerous plant extract has been added to ram semen diluent to improve cryopreserved semen quality (Ren et al. 2021). Moreover, other studies have tried to improve cryopreserved ram semen through supplementing long chain polyunsaturated fatty acid sources such as fish oil successfully (Taghilou et al. 2017). However, supplementing fish oil might increase human-animals food competition hence can lead to the failure to feed highly growing population as projected by (FAO 2022). Therefore, this necessitate the need to harness alternative source of long-chain polyunsaturated fatty acids (LCPUFAs) particularly the plant-based precursors such as the flaxseed oil.

Flaxseed oil has a high concentration of alpha-linolenic acid (58%) that is broken down *de novo* to generate docosahexaenoic acid, which is essential for testicular function (Francois et al. 2003; Zanussi et al. 2019; Ngcobo et al. 2021). Docosahexaenoic acid (DHA) and eicosapentaenoic (EPA) synthesised de novo through supplementing flaxseed oil alter the sperm cell membrane structure, resulting in a fluid sperm membrane and participation in cell response mediated by protein (Tang et al. 2019). Nevertheless, supplementing with flaxseed oil require a natural antioxidant to balance the influence. Ascorbic acid, is a naturally occurring antioxidant that has been shown to boost sperm quality and eliminate free radicals (Attia et al. 2020) when fed with the long chain polyunsaturated fatty acid processors (Singh et al. 2021). Despite intensive work done to investigate influence of flaxseed oil on fresh semen quality from rams (Ngcobo et al. 2022, 2023), there are few (if any) studies that evaluated the influence of dietary inclusion of flaxseed oil and ascorbic acid on cryopreserved sperm quality and in vitro fertility. Therefore, determining how flaxseed oil and ascorbic acid affects cryopreserved sperm quality, and in vitro fertilization would pave the way for improved sperm survival and fertility. Thus, the goal of this study was to determine the effect of flaxseed oil and ascorbic acid on cryopreserved sperm quality, and in vitro fertilization.

## Materials and methods

### Ethic statement

The study procedures were authorized by the Agricultural Research Council (APAEC 2019/33) and the Tshwane University of Technology Animal Research Ethics Committee (AREC2020/05/001).

### Experimental site

The current study was carried out at the South African Agricultural Research Council (ARC), Irene, Animal Production (ARC, Irene). This region is located between 25^o^53’59.6” South latitude and 28^o^12’51.6” East longitude. In this region, sheep are reproductive active during late autumn to winter season normally between March – May (Malejane et al. 2014). Animals were kept in a same environmental condition with free access to water but no access to the grass pasture throughout the study.

### Experimental animals

Study rams were housed in 5 trial pens as per treatment diets to limit stress while allowing socialization among rams. In this study, 22 matured indigenous South African rams (age = 6 years, average body weight = 64.4 ± 1.6) were used. They were then fed in the morning every day with water provided *ad-libitum*. Twenty-two study rams were randomly allocated 5 diets (neg. cont.), pos. cont., FLO, ASA and FLO + ASA). The neg. cont. diet had 4 rams pos. cont. had 4 rams FLO had 5 rams ASA had 4 rams and FLO + ASA diet had 5 rams. The treatment diets were formulated as described by the National Research Council, 1985. Following the formulation of treatment diets, FLO diet was top mixed with 5% flaxseed oil, ASA diet was mixed with 4% ascorbic acid and FLO + ASA diet was mixed with 5% flaxseed oil and 4% ascorbic acid. Moreover, negative control was the diet in use at the Agricultural Research Council, Irene made of eragrostis curvular hay and 200 gram of pellets per ram/day whereas, the positive control was the diet formulated according to NRC (1985) without flaxseed oil and ascorbic acid. Thereafter, the formulated diets were then sampled and taken to South African National Accreditation System (SANAS) accredited laboratory for feed composition analysis can be found in Table 1. Feed ingredients used in this study included Eragrostis curvular hay, soya bean, bran, limestone and P12 bio mineral.

**Table 1:** Proximate analysis of the treatment diets (Ngcobo et al. 2023)

Proximate analysis	Neg. Cont.	Pos. Cont.	FLO	ASA	FLO + ASA
Dry matter	88.23	88.93	92.20	90.42	93.52
Ash	5.32	12.36	5.00	5.49	4.75
Crude Protein	10.73	10.36	10.74	10.29	10.59
Either Extract	2.03	2.16	19.79	1.70	24.29
Crude Fiber	29.01	21.71	25.90	28.71	20.95

NB – neg. cont. (standard diet), pos. cont. (basal diet), FLO (basal diet top dressed with 5% flaxseed oil), ASA (basal diet top dressed with 4% Ascorbic Acid), FLO + ASA (basal diet top dressed with both 5% flaxseed oil + 4% Ascorbic Acid).

### Experimental design

#### Semen collection and evaluation

Semen was collected by the artificial vagina following a 3 weeks training period and 60 days of supplementing with five study diets during breeding season (March to June, 2022). Semen was collected twice a week to maintain 2 days resting period between collection for 3 months (March, April, May and June) giving us a total of 704 ejaculates collected from all rams. Computer-aided sperm analysis (CASA), Sperm Class Analyzer^®^ system (SCA) 5.0 version (Microptic, Barcelona, Spain) at a magnification of 10x (Nikon, Japan) and mounted with a warm plate (37 °C) was used to assess sperm motility parameters following semen cryopreservation, before in vitro fertilization. Sperm cell parameters evaluated using CASA included total motility, non-progressive motility, progressive motility, rapid, medium and slow motility, curvilinear velocity, average path velocity, straight line velocity, linearity, straight-line and wobble. Concoction of sperm and tris-swim up was pipetted to the warmed microscope slide (76 × 26 × 1 mm, Germany) and gently enclosed with cover slip (22 × 22 mm, Germany) for sperm motility parameters. A total of four fields containing ± 200 sperm were randomly selected per field and analysed using Computer-Aided sperm analysis (CASA). Sperm plasma membrane was evaluated using the hypo-osmotic swelling test (HOST) as reported by (Chatiza et al. 2018) with a few modifications. In brief, 100µL cryopreserved semen was mixed with 500µL HOST (sodium citrate = 0.3675, fructose = 0.6755 in 50 ml Falcon tube) and incubated at 37 °C for 30 min. Following 30 min of co-incubation, 10µL of the concoction was pipetted and gently distributed on the microscope slide, which was then examined under the microscope as follows: 1 - intact sperm cells with a curved tail, 2 - non-intact/damaged sperm cells with a straight tail.

Sperm morphology and viability was assessed following cryopreservation, before in vitro fertilization. Cryopreserved semen samples were mixed with the eosin-nigrosin (5% Eosin and 10% nigrosin) stain (Ondersterpoort Faculty of Veterinary Science’ Pharmacy, South Africa) at a ratio of 5µL:20µL. The concoction was distributed on the microscope slide gently and dried at a room temperature for 24 h before evaluation. After 24 h, immersion oil was smeared and the total of 200 sperm cells were counted under the Olympus microscope (Olympus microscope, BX51, Japan) magnificent (UPlan FLN, 100x/1.30 011 /0.17 / FN26.5). Sperm abnormalities were classified as primary, secondary and tertiary abnormalities. Primary abnormalities are the abnormalities occurring during spermatogenesis including double head and double tail. Secondary abnormalities are the abnormalities occurring during ejaculation and these includes: proximal droplets and distal droplets. Tertiary abnormalities are those occurring due to the in vitro sperm handling and includes: coiled tail and bent tail.

#### Experiment 1: semen cryopreservation

Semen samples were then evaluated for sperm motility and only semen samples with between 80 and 90% total motility was cryopreserved. Tris-egg yolk-based extender was used to extend semen samples. The composition of the Tris-egg yolk-based extender chemical composition were: tris, 2.11 g; citric acid, 0.68; monohydrate glucose, 0.5; gentamycin sulphate, 0.05; egg yolk 10mL and deionized water of 40mL (Salamon and Maxwell 2000). Glycerol (9mL) was added on the second fraction. After four hours of equilibration at 5˚C, semen samples were loaded to 0,25mL polyvinyl alcohol semen straws using different colours as per treatment diets.

Programmable freezer (CBS Freezer 2100 series, Laboratory consumables & chemical suppliers, America) was used to cryopreserve semen with the following rate settings: step 1 – cooling rate 0.00 (C/min), demand temperature 5˚C and the holding time of 0.00 min, step 2: cooling rate of 0.08˚C/min, demand temperature of 4˚C, holding time of 5 min, step 3: cooling rate 6˚C/min, demand temperature of -130˚C with the holding time of 10 min. Following cryopreservation with the programmable freezer (CBS Freezer 2100 series, Laboratory consumables & chemical suppliers, America), semen straws were dipped to the Styrofoam box (Plastilion packaging, South Africa) with liquid nitrogen. Semen straws were then loaded in the cannisters and kept for 6 months. On the day of in vitro fertilization, semen straws were thawed using warm water (37 ˚C) for 1 min and evaluated using CASA before use either for in vitro fertilization.

#### Experiment 2: fertility of cryopreserved semen

##### Sheep ovary collection

Sheep ovaries were taken within 2 h after slaughter from a local slaughterhouse (Cavalier Foods (Pty) Ltd), Gauteng Province and conveyed to the laboratory in a thermos flask containing 0.9% buffered saline water (Adcock Ingram critical care, SA) at 37 °C. Up on arrival at the laboratory ovaries were rinsed with clean saline water to eliminate blood contamination from donor sheep and sprayed with 75% ethanol absolute (Laboratory Consumables and Chemicals supply cc, SA).

##### Oocytes retrieval and grading

Ovaries were selected and put on a petri dish (Falcon 1008, USA) with modified Dulbecco’s phosphate buffered saline (mDPBS), processed through slicing method by chopping ovaries into tiny pieces using a surgical blade (Hi-Careint, Japan), and transferred into a 50 mL falcon tube (Plastpro Scientific, SA) containing wash medium^®^ (Bioscience, UK). Follicular fluid was transferred into a graded petri dish (Carbi, USA), and COCs were selected from the mixture using a microscope (Olympus BX71, Philippines) at a magnification of 10x/H22.

The oocytes were categorised as grade A, B, or C, with grade A oocytes containing several entire layers of cumulus cells and uniform cytoplasm. Grade B oocytes had partial layers of cumulus cells and homogenous cytoplasm, while Grade C oocytes had no cumulus cells. Oocytes retrieved in grades A, B, and C was counted, however only grade A was used for In Vitro Maturation (IVM), In Vitro Fertilization (IVF) and In Vitro Culture (IVC).

##### In vitro maturation of oocytes

After collecting, processing, and grading all oocytes, a total of 500 L Brackett and Olifant (BO) wash medium^®^ (Bioscience, UK) washed grade A oocytes three times. These oocytes were in vitro matured in 4-well pans with 500 L BO-IVM^®^ (Bioscience, UK) enriched with low glucose, gonadotrophic hormones, and gentamycin. Following that, the oocytes were incubated for 24 h at 38.5 OC with 5% CO_2_, 5% oxygen (O_2_), and 100% humidity.

##### Polar body evaluation

After 24 h, a portion (25%) of matured oocytes were out of the incubator and vortexed for 3 min in 200µL while the remaining oocytes (75%) were kept for IVF purposes. They were then placed on a petri dish with 3 mL of BO wash media for polar body extrusion. The Oosight Imaging System (Hamilton Thorne) connected to the microscope Olympus 1 × 71 (New York microscope Co, USA) at 20x/0.45 Rc2 magnification was used to examine the vortexed oocytes for polar body extrusion between the zona pellucida and the cytoplasmic space.

##### Preparation of matured oocytes for in vitro fertilization

Oocytes matured in BO IVM^®^ media were washed five times in 50µL BO-IVF medium (IVF Bioscience, UK). These oocytes were then placed into 100µL fertilization micro drops coated with 3 mL mineral oil.

##### Thawing of frozen semen straw and sperm capacitation

In vitro fertilization was achieved by utilizing cryopreserved sperm collected from the experimental rams according to their treatment diets (neg. cont., pos. cont., FLO, ASA, and FLO + ASA). (Ngcobo et al. 2023). For thawing of semen, the frozen straws from each diet were held in the air for ten seconds before being dropped into 37 °C water for a min. The straws were dried and sliced on both sealed ends, and the contents were emptied into 15 mL falcon^®^ tubes (Nest Biotechnology Co, China). Prior fertilization, sperm (5µL) distributed in a warm microscope slide (Labocare, UK) to examine the sperm motility parameters under the CASA system. The BO semen prep^®^ media was used to select sperm (IVF Bioscience, UK). The contents of the frozen thawed straws were diluted with 4 mL of prewarmed semen preparation medium in 15 mL tubes and centrifuged at 1500 g force for 5 min. Following centrifugation, the supernatant was removed, and sperm suspension remained in a 4 mL tube; then, fresh warmed BO semen prep^®^ medium was added, and the combination was resuspended and centrifuged. Following the second centrifugation, the supernatant was removed until the same volume of 350–700µL sperm suspension remained; the pellet in this volume with a concentration of 2.0 × 106/mL was diluted with semen prep medium, suspended, and utilized for IVF.

##### In vitro fertilization of matured oocytes

Oocytes remained from polar body evaluation were fertilized with their respective dietary cryopreserved semen. Twenty-five (25%) of these oocytes were incubated for 6 h for pronucleus evaluation, while the remaining 50 oocytes were incubated for 18 h at 38.5 ^O^C with 5% CO_2_, 5% O_2_, and 100% humidity for IVC purposes.

##### Preparation of presumptive zygotes for staining and pronucleus evaluation

Following 6 h of IVF, presumptive zygotes were extracted from fertilization drops and cumulus cells were extracted by vortexing in 200 L of pre-warmed wash media (Bioscience, UK) for one min and 30 s. The zygotes were then washed in wash medium before being stained with a Hoechst solution.

Four drops of Petroleum jelly (Vaseline, Unilever South Africa) were placed in a square pattern around a tiny slide. Presumptive zygotes were moved to a glass slide. Hoechst solution (2–10µL) (stock 2) of was applied to the sides of the cover slip up until the entire surface underneath the slip was coated. This was done with caution to avoid washing the presumptive zygotes out of the slide. A cover slip (Labocare, UK) was gently pushed over the microscopic slide until reaching the presumptive zygotes drop. The cover slip’s ends were immediately sealed with colourless nail polish. Prepared slides dried for 2 h in a dark area before examination using an inverted microscope Olympus 1 × 71 (New York microscopy Co, USA) at 20x/0.45 Rc2 magnification with a UV filter. After 2 h, the slides were examined for pronucleus status.

##### In vitro culture of presumptive zygotes

For IVC purposes, presumptive zygotes were transferred from fertilization drops into pre-warmed wash medium^®^, and vortexed for 1 min 10 s for removal of cumulus cells. Presumptive zygotes were washed five times in 50µL of pre-warmed BO IVC^®^ medium (Bioscience, UK) drops and moved into the new 100µL BO IVC^®^ drops of the same medium that had been coated with mineral oil. These were then cultured for 48 h in modular chamber with 5% O_2_ and 5% CO_2_ mixed gas added for a min and incubated for 48 h. After 48 h of culture, the sperm-exposed presumptive zygotes were examined for cleavage rate (day two of IVC).

## Statistical analysis

Data (sperm parameters and in vitro fertility parameters) was exposed to a General Linear Model (GLM) in Minitab 17^®^. Pearson’s correlation coefficient was utilized to correlate cryopreserved sperm quality and in vitro fertility parameters. The student Least Significant Difference Test was used to separate the treatment means, and differences were accepted when the *p*-value was less than 0.05.

## Results

### Semen cryopreservation

The current study investigated the effect of flaxseed oil and Ascorbic acid on the cryopreserved semen quality and fertility and the findings are shown in Table 2. The progressive motility was higher in FLO + ASA treated group than neg. cont., pos. cont., FLO and ASA. The total motility was higher (*p* < 0.05) in the FLO + ASA than the neg. cont., pos. cont., FLO and ASA. Static motility was higher in the neg. cont., than that in the FLO + ASA, ASA, FLO and the pos. cont. groups.

**Table 2:** Effect of treatment diets on cryopreserved semen quality following dietary flaxseed oil and ascorbic acid

Parameters	Neg. Cont.	Pos. Cont.	FLO	ASA	FLO + ASA
Progressive motility	19.97 ± 1.87^c^	19.80 ± 1.87^c^	28.19 ± 1.87^b^	27.02 ± 1.87^b^	36.33 ± 1.87^a^
None-progressive motility	29.36 ± 2.24^c^	44.20 ± 2.24^b^	52.51 ± 2.24^a^	52.32 ± 2.24^a^	51.91 ± 2.24^a^
Total motility	49.33 ± 2.24^d^	64.00 ± 2.24^c^	80.70 ± 2.24^b^	79.34 ± 2.24^b^	88.24 ± 2.24^a^
Rapid motility	14.07 ± 1.74^bc^	13.20 ± 1.74^c^	18.92 ± 1.74^b^	17.73 ± 1.74^bc^	27.52 ± 1.74^a^
Medium motility	7.73 ± 1.05^b^	9.94 ± 1.05^b^	14.10 ± 1.05^a^	14.93 ± 1.05^a^	16.16 ± 1.05^a^
Slow motility	27.53 ± 2.12^c^	40.86 ± 2.12^b^	47.68 ± 2.12^a^	46.68 ± 2.12a^b^	44.54 ± 2.12^ab^
**Sperm plasma membrane integrity (%)**					
Intact Sperm	52.33 ± 2.08^c^	58.13 ± 2.08^c^	70.87 ± 2.08^ab^	69.67 ± 2.08^b^	75.67 ± 2.08^a^
Sperm viability (%)					
Live Sperm	65.00 ± 1.37^b^	60.93 ± 1.37^c^	74.20 ± 1.37^a^	72.27 ± 1.37^a^	75.73 ± 1.37^a^
Sperm abnormalities (%)					
Primary	13.00 ± 0.96^a^	7.07 ± 0.96^b^	4.20 ± 0.96^c^	4.27 ± 0.96^c^	4.27 ± 0.96^c^
Secondary	7.13 ± 0.89^a^	5.47 ± 0.89^ab^	3.67 ± 0.89^b^	6.47 ± 0.89^a^	3.47 ± 0.89^b^
Tertiary	14.80 ± 1.85^b^	20.13 ± 1.85^a^	16.93 ± 1.85^ab^	13.80 ± 1.85^b^	17.47 ± 1.85^ab^

^a, b, c^ Means with different superscripts within the raw, differ significantly (*p* < 0.5). NB – neg. cont. (standard diet), pos. cont. (basal diet), FLO (basal diet top dressed with 5% flaxseed oil), ASA (basal diet top dressed with 4% Ascorbic Acid), FLO + ASA (basal diet top dressed with both 5% flaxseed oil + 4% Ascorbic Acid)

Rapid motility was higher (*p* < 0.05) in the FLO + ASA groups when compared with that of ASA, FLO, positive and the neg. cont. On the other hand, Medium motility in the FA + ASA and ASA were higher (*p* < 0.05) than that of neg. cont. and the pos. cont. Slow motility was higher in the FLO + ASA, ASA, FLO than the neg. cont. group. The dietary treatment effect was also investigated through evaluating the sperm plasma membrane, viability and sperm abnormalities in cryopreserved semen following dietary supplementation of flaxseed oil and Ascorbic acid and the results are obtainable in Table 2. The effect of treatment diets (neg. cont., pos. cont., FLO, ASA and FLO + ASA) on sperm plasma membrane, viability and abnormalities were also evaluated and the results are obtainable in Table 2. There was a higher (*p* < 0.05) intact sperm cells in FLO + ASA and FLO (70.87 ± 2.08) than that of the neg. cont. and the pos. cont. Intact sperm cells in ASA could not differ (*p* > 0.05) to FLO diet.

For sperm viability, there was a greater (*p* < 0.05) live sperm cells following supplementing the FLO + ASA, ASA and the FLO than that of the positive and the neg. cont. Primary abnormalities could not differ (*p* > 0.05) among FLO + ASA, ASA and FLO however, were lower than that of the pos. cont. and the neg. cont. Secondary sperm abnormalities were higher in the ASA, neg. cont. and the pos. cont. than that from FLO + ASA and the FLO. Tertiary sperm abnormalities were higher in the pos. cont., than the neg. cont., FLO, ASA and FLO + ASA.

### Fertility of cryopreserved semen

**Fig. 1:**
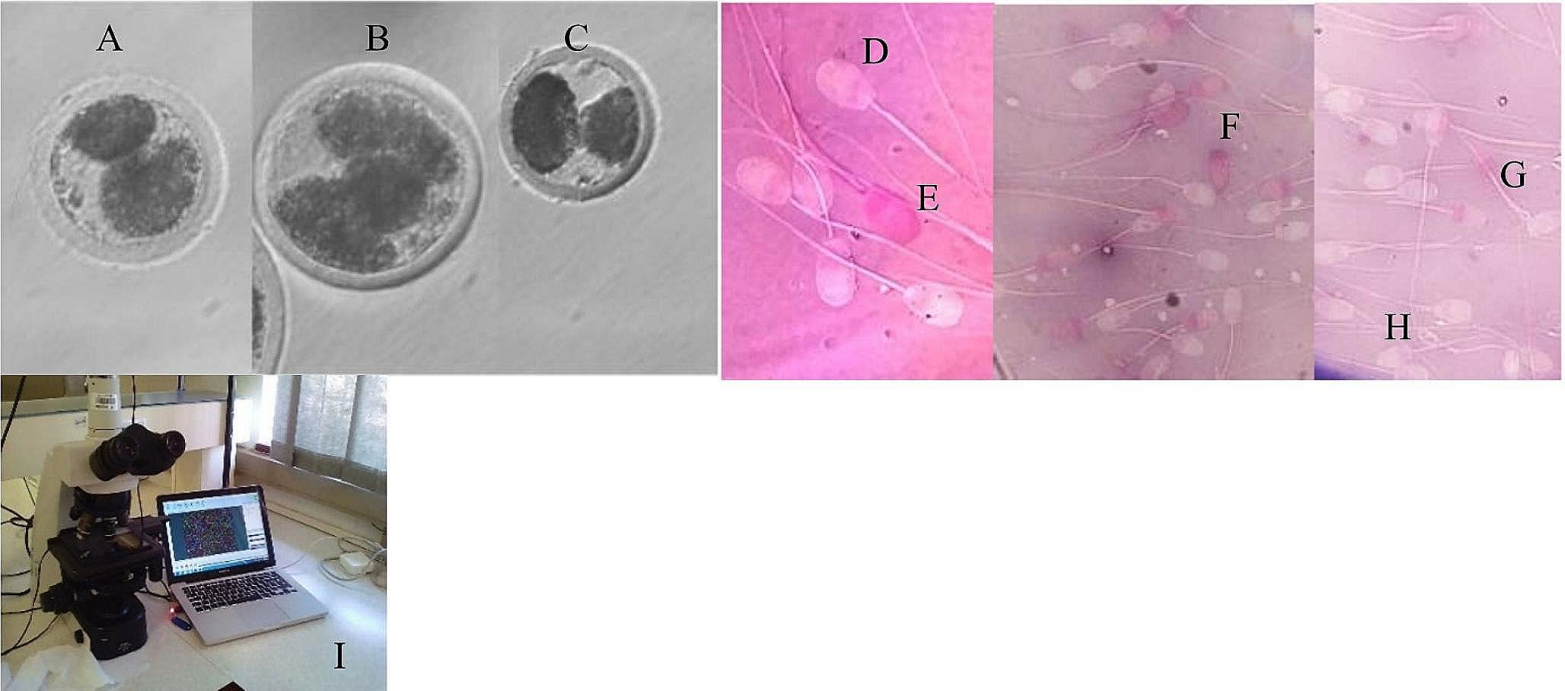
Illustrate in vitro culture (**A-C** – in vitro culture), sperm viability (**D** – live sperm, **E** – dead sperm), some sperm abnormalities (**F** – detached sperm/tailless sperm, **G** – bent tail and **H** – coiled tail) and **I** - Computer-Aided Sperm analyser

The pronucleus evaluation following the use of cryopreserved semen (Fig. 1) was conducted and the results are obtainable in Table 3. There were higher (*p* < 0.05) none PN oocytes in the neg. cont., pos. cont., FLO, and ASA groups than that of FLO + ASA group. No differences observed for the 1 PN, 2PN and > 2PN among all treatment groups (neg. cont., pos. cont., ASA, FLO and FLO + ASA). However, the total fertilization was higher (*p* < 0.05) in the FLO + ASA group than the neg. cont., pos. cont. and ASA treated groups.

**Table 3:** Depicts the dietary effect on in vitro fertilization

Parameters	Neg. Cont.	Pos. Cont.	FLO	ASA	FLO + ASA
**In vitro fertilization rate (%)**					
No. of IVF oocytes	8.33 ± 0.66^ab^	9.17 ± 0.70^ab^	7.36 ± 0.60^b^	9.06 ± 0.77^ab^	9.56 ± 0.77^a^
None PN%	59.61 ± 6.43^a^	62.62 ± 6.82^a^	47.47 ± 5.80^ab^	56.01 ± 6.43^a^	34.02 ± 6.82^b^
1 PN%	21.83 ± 4.28^a^	24.60 ± 4.54^a^	28.59 ± 3.86^a^	20.93 ± 4.28^a^	34.58 ± 5.02^a^
2 PN%	7.08 ± 5.07^a^	7.78 ± 5.38^a^	14.50 ± 4.57^a^	18.44 ± 5.07^a^	23.15 ± 5.95^a^
> 2 PN%	13.15 ± 4.14^a^	7.38 ± 4.39^a^	8.39 ± 3.73^a^	4.63 ± 4.14^a^	8.25 ± 4.86^a^
TF%	42.06 ± 6.30^b^	39.76 ± 6.68^b^	51.49 ± 5.68^ab^	43.99 ± 6.30^b^	65.98 ± 7.39^a^
**In vitro cleavage rate (%)**					
Lysed	0	0	0	0	0
Oocytes cleaved	8.67 ± 0.43^a^	9.06 ± 0.40^a^	9.50 ± 0.43^a^	9.50 ± 0.43^a^	9.50 ± 0.43^a^
1Cell%	71.62 ± 6.50^ab^	75.60 ± 6.13^a^	32.86 ± 6.50^d^	52.82 ± 6.50^bc^	33.81 ± 6.50^cd^
2-4Cell%	28.38 ± 5.55^c^	23.01 ± 5.24^c^	63.81 ± 5.55^a^	45.51 ± 5.55^b^	66.19 ± 5.55^a^
6-8Cell%	0	1.39 ± 1.53^a^	3.33 ± 1. ±63^a^	1.67 ± 1.63^a^	0
Total cleavage%	28.38 ± 6.50^cd^	24.40 ± 6.13^d^	67.14 ± 6.50^a^	47.18 ± 6.50^bc^	66.19 ± 6.50^ab^

^a, b^ Means with different superscripts within the raw differ significantly (*p* < 0.05). neg. cont. (standard diet), pos. cont. (basal diet), FLO (basal diet top dressed with 5% flaxseed oil), ASA (basal diet top dressed with 4% Ascorbic Acid), FLO + ASA (basal diet top dressed with both 5% flaxseed oil + 4% ascorbic acid). IVF – In vitro fertilization, 1PN – 1 Pronucleus, 2PN – 2 Pronucleus, >2PN – Polyspermy, TF – Total Fertilization

The dietary effects (neg. cont., pos. cont., FLO, ASA and FLO + ASA) on the cleavage rate (Fig. 1) was investigated and the results can be found in Table 3. The number of presumptive zygotes cleaved was the same for all the treatment diets. The FLO recorded the lowest 1cell percentage when compared to neg. cont., pos. cont., and ASA however not different from FLO + ASA. The FLO and FLO + ASA recorded a significant higher percentage of the presumptive zygotes (2-4cells) when compared to neg. cont., pos. cont., and ASA. No significant difference recorded on the presumptive zygotes with 6-8cell on the pos. cont., FLO, and ASA nevertheless none were recorded for the neg. cont. and the FLO + ASA. Noteworthy, the FLO (67.14%) and the FLO + ASA recorded greater total cleavage rate in comparison with the neg. cont., pos. cont., and ASA.

The relationship between cryopreserved sperm parameters and the IVF, IVC and the conception rate were correlated using the Pearson’s correlation coefficient and the results are obtainable in Table 4. Two pronucleus had a medium relationship with progressive motility (r^2^ = 0.331), non-progressive motility (r^2^ = 0.303), total motility (r^2^ = 0.418) and medium motility (r^2^ = 0.331). A weak relationship was observed between 2 pronucleus and rapid motility (r^2^ = 0.292). Total fertilization rate had a medium significant (*p* < 0.001) medium correlation with the progressive motility (r^2^ = 0.435), total motility (r^2^ = 0.447) and rapid motility (r^2^ = 0.409). For in vitro culture, there was a significantly (*p* < 0.001) strong relationship between 2 and 4 cell and progressive motility (r^2^ = 0.558) and rapid motility (r^2^ = 0.594) whereas, 2–4 cell had a significantly (*p* < 0.001) medium relationship with the non-progressive motility and the weaker relationship with slow motility (r^2^ = 0.358). The total in vitro culture rate had a significantly (*p* < 0.001) strong correlation with the total motility (r^2^ = 0.610) and rapid motility (r^2^ = 0.539). Moreover, there was a substantial (p 0.001) medium association between in vitro culture and increasing motility (r2 = 0.422). The medium and slow motility had a significantly (*p* < 0.001) weak correlation with total in vitro culture (r^2^ = 0.302, r^2^ = 0.377), respectively. Conception rate was not significantly (*p* > 0.05) correlated to any of the sperm cell parameters following cryopreserved processes.

**Table 4:** Pearson correlation between cryopreserved sperm parameters, in vitro fertilization, in vitro culture and the conception rate

Parameters	1PN	2PN	> 2PN	TF	1 Cell	2–4 Cell	6–8 Cell	TC	Progressive motility	None-progressive motility	Total motility	Rapid motility	Medium motility	Slow motility
1PN	1.00													
2PN	0.170NS	1.00												
> 2PN	−0.419**	−0.371NS	1.00											
TF	0.620***	0.698***	−0.011NS	1.00										
1 Cell	−0.279*	−0.358NS	0.080NS	−0.440***	1.00									
2–4 Cell	0.321**	0.363**	−0.124NS	0.449***	−0.987***	1.00								
6–8 Cell	−0.133NS	0.101NS	0.216NS	0.110NS	−0.435***	0.283*	1.00							
TC	0.279*	0.358**	−0.080NS	0.440***	−1.000NS	0.987***	0.435***	1.00						
Progressive motility	0.229NS	0.331*	0.008NS	0.435***	−0.508***	0.558***	−0.044NS	0.508***	1.00					
Nnoe-progressive motility	0.258NS	0.303*	−0.287*	0.259NS	−0.422***	0.414***	0.201NS	0.422***	0.132NS	1.00				
Total motility	0.324*	0.418**	−0.205NS	0.447***	−0.610***	−0.610***	0.091NS	0.610***	0.690***	0.809***	1.00			
Rapid motility	0.260NS	0.292*	−0.431***	0.409***	−0.539***	0.594***	−0.111NS	0.539***	0.948***	0.096NS	0.632***	1.00		
Medium motility	0.126NS	0.331*	−0.404***	0.382***	−0.302*	0.329*	−0.037NS	0.302**	0.660***	0.416***	0.695***	0.439***	1.00	
Slow motility	0.237NS	0.259NS	−0.206NS	0.189NS	−0.377**	0.358**	0.244NS	0.377**	0.012NS	0.976***	0.720***	−0.013NS	0.257NS	1.00
Conception rate	0.009NS	−0.129NS	0.079NS	−0.049NS	0.105NS	−0.120NS	0.047NS	−0.105NS	−172NS	0.028NS	−0.082NS	−0.180NS	−0.044	0.036NS

NS – Non-Significant (*p* > 0.05), * *p* < 0.05, ** *p* < 0.01, *** *p* < 0.001. PN – Pronucleus, TF – Total Fertilization, TC – Total Cleavage

## Discussion

### Semen cryopreservation

The objective of the study was to investigate the dietary effects of flaxseed oil and Ascorbic acid on the quality and fertility of cryopreserved sperm. The dietary supplementation with flaxseed oil in South African rams’ diets can improve fresh semen quality during breeding season (Ngcobo et al. 2023) and throughout the years (Ngcobo et al. 2023). As a results, dietary inclusion of dietary omega n-3 sources to improve reproduction performance in sheep is of a great interest to implement assisted reproductive technologies in the conservation program (Kargar et al. 2017). Cryopreservation techniques increase plasma membrane fluidity-permeability and reactive oxygen species generation (Peris-Frau et al. 2020). Moreover, molecular investigations demonstrated that sperm plasma membrane and cholesterol levels changed after cryopreservation (Peris-Frau et al. 2020).

Dietary flaxseed oil had an influence on cryopreserved sperm quality where, flaxseed and Ascorbic acid supplemented group performed better than the negative and the pos. cont. for progressive, non-progressive, total motility and rapid motility. Similar outcomes were observed by (Kumar et al. 2021) in growing male lambs and in bucks out of breeding season (Souza et al. 2019) rams out of breeding season (Ngcobo et al. 2023) and in bulls (Moallem et al. 2015). Cryopreserved sperm quality improvement observed in this study can be justified by the docosahexaenoic acid since it is a main phospholipid coating the sperm plasm membrane (Martínez-Soto et al. 2016), making up to 30% of esterified fatty acids in the phospholipids and 73%of all polyunsaturated fatty acids (Aké-Villanueva et al. 2019). Flaxseed oil contain 58% alpha-linolenic acid, which is an essential antioxidant known to improve animal health and production (Khan 2015) and the general animal reproductive health (Perumal et al. 2019). Alpha-linolenic acid is a docosahexaenoic acid (DHA) precursor synthesized with the aid of ∆5 and ∆6-desaturase enzymes (Nandi et al. 2019; Collodel et al. 2020). Apart from long-term conservation of sperm cells, cryopreservation reduces the cost of keeping rams in the farm and the prevention of genetic drift (Barbas and Mascarenhas 2009). Furthermore, cryopreservation simplifies worldwide sperm commerce and allows for long-term preservation of superior sire sperm (Peña et al. 2011). Nevertheless, cryopreservation promotes numerous cell damages including reduction in sperm membrane fluidity, lipid, and protein organization (Fernandes et al. 2021).

However, ram sperm cells are sensitive to cryopreservation processes in comparison to other domestic animals (Curry 2000) due to higher sensitivity to low temperature (Salamon and Maxwell 2000) and low cryotolerance hence, less than 30% of sperm cells remain undamaged following cryopreservation (Salamon and Maxwell 2000). This cryo-damages are informed by oxidative, osmotic and thermal stresses that are harmful to the sperm cells and can cause poor conception rate (Tariq et al. 2015). In the current study, when the influence of diet was introduced, FLO + ASA and FLO had higher intact plasma membrane integrity when compared to the negative and the pos. cont. following cryopreservation. It is generally established that cryopreservation alters sperm cell motility, causes membrane modifications affecting sperm capacitation and acrosome response, and reduces sperm viability (Martin et al. 2004). Furthermore, seminal plasma contains proteins that shield sperm against cold shock damage and increase sperm viability (Pérez-Pé et al. 2001). Among these proteins tripeptidyl-peptidase, heat-shock protein 90 and chaperonin-containing t-complex polypeptide 1 are the main protein regulating cryo-capacitation, protect sperm against oxidative stress and stabilizes sperm membrane through an efficient protein folding (Wang et al. 2013; Rickard et al. 2015). These findings matched those published by (Mandiki et al. 1998) in Texel, Suffolk, and Ile-de-France rams. However, their results might be justified by the season and the age of rams (Belkhiri et al. 2017). Noteworthy, the current study revealed that, rams fed FA + ASA, ASA and FLO had significant good sperm viability through higher live sperm cells and lower dead sperm cells post-thawing. Sperm viability and plasma membrane integrity has been reported as the strong indicators of sperm functions (Peña et al. 2011). On the opposite hand, the current study observed significant lower sperm abnormalities in the dietary supplemented groups especially for primary abnormalities.

Sperm abnormalities were then classified into primary, secondary and tertiary sperm abnormalities (Ngcobo et al. 2023). Primary sperm abnormalities happen during spermatogenesis, secondary sperm abnormalities happen after ejaculation, and tertiary abnormalities happen during invitro sperm handling (Ngcobo et al. 2023). Sertoli cells in the testicles are active in conversion of 18 and 20 carbon omega n-3 into 22 carbon LCPUFAS (68) due to their high concentration of desaturase mRNA and elongate enzymes (Souza et al. 2019). Moreover, DHA provides essential sperm membrane fluidity and mediate sperm cell response to protein (Nandi et al. 2019) and supports the role of astrocyte, retina pigment epithelial cells and Sertoli cells in the testicles (Sæther et al. 2007). High polyunsaturated fatty acid and low antioxidant capacity in ram sperm cells lead to the build-up of reactive oxygen species (ROS) during cryopreservation. Oxidative stress can be regulated by inclusion of antioxidants during cryopreservation (Tariq et al. 2015). Nevertheless, cryopreservation still produces high ROS, osmotic stress, spermatic DNA damage, membrane destabilization and dysfunction (Vichas et al. 2018).

### Fertility of cryopreserved semen

Following evaluation of cryopreserved sperm quality after dietary supplementation flaxseed oil and Ascorbic acid, sperm cell was tested for in in vitro fertilization. Noteworthy, FLO + ASA had a highest total fertilization although could not differ with that observed in FLO. Currently, in our best knowledge there are less studies that evaluated the influence of dietary flaxseed oil on in vitro fertilization rate. Nevertheless, it is well known that only ± 60% of sperm cells remain viable and motile after cryopreservation (Lv et al. 2019) and only ± 30% remain biological functional (Salamon and Maxwell 2000). Numerous domestic animals are born following the use of assisted reproductive biotechnologies (Viana 2020.). Slaughtered ovary donor of a superior genetic material can reduce the generation interval by ± 3–6 months (Amiridis and Cseh 2012) hence, cryo-gene bank is a vital strategy in conservation of farm animal genetic material (Leroy et al. 2019). However, generally there is a decline in ovine embryo production with non-reported in Africa (Viana 2020). As a result, the findings of this work can make a significant contribution to the development of effective cryo-gene banks for the preservation of embryos of high genetic value and from endangered species.

In vitro culture result followed a similar trend to that of in vitro fertilization. In brief, groups supplement with FLO + ASA and FLO had the highest total cleavage in comparison to negative and the pos. cont. The neg. cont., and the pos. cont. were both not supplemented with flaxseed oil and it is known that low DHA in the sperm cell has been associated with the infertility (Moallem et al. 2015). Furthermore, dietary supplementation of omega n-3 sources is proven to increase sperm and subsequent fertilization rate motility (Ahmad et al. 2019) thus the great interest in dietary inclusion of dietary omega n-3 precursors on domestic livestock diets (Díaz et al. 2017). Despite other numerous factors affecting the efficient of in vitro embryo production, semen quality (fresh or frozen) and the selection of semen has been major factors (Falchi et al. 2018).

Sperm cells with defective DNA can fertilize, however embryo development can be interrupted once the embryo genome is active at the 4 or 8 cell stage due to improper paternal gene transcription (Kumar and Naqvi 2014). Therefore, there was a need to evaluate the conception rate following dietary supplementation of flaxseed oil and Ascorbic acid. Dietary (FLO and FLO + ASA) treated groups performed better than other groups. Shahid et al. (2019), observed similar results in chickens and suggested that flaxseed oil enriched diets change the sperm PUFAs’ mostly during the decreasing phase of annual reproductive period. Moreover, in cows supplementing flaxseed oil Improved pregnancy rate and reduced uterine PGF2α secretion and decreased sensitivity of the corpus luteal to PGF2α during important stage of embryonic development (Nazir et al. 2013).

In conclusion, dietary inclusion of FLO + ASA improve frozen thawed sperm quality, total in vitro fertilization and total cleavage rate. Therefore, the FLO can be used to improve cryopreserved sperm quality. Furthermore, the progressive, total and rapid motility play a crucial in the in vitro fertilization rate. These results may pave a way to disseminate superior genetic material and improve South African indigenous sheep population through in vitro embryo production.

## Data Availability

This study’s intellectual property is owned by TUT and the ARC, Irene (ARC). However, the data is available from the corresponding author when requested.
